# Multiple UDP-Glucuronosyltransferase and Sulfotransferase Enzymes are Responsible for the Metabolism of Verproside in Human Liver Preparations

**DOI:** 10.3390/molecules22040670

**Published:** 2017-04-22

**Authors:** Ju-Hyun Kim, Deok-Kyu Hwang, Ju-Yeon Moon, Yongnam Lee, Ji Seok Yoo, Dae Hee Shin, Hye Suk Lee

**Affiliations:** 1 Drug Metabolism & Bioanalysis Laboratory, College of Pharmacy, The Catholic University of Korea, Bucheon 14462, Korea; jhyunkim@catholic.ac.kr (J.-H.K); myhdg@naver.com (D.-K.H.); jy_moon@catholic.ac.kr (J.-Y.M); 2 Central R&D Institute, YUNGJIN PHARM. CO., LTD., Suwon 16229, Korea; nami0209@yungjin.co.kr (Y.L.); jsyoo@yungjin.co.kr (J.S.Y.); jkyk58@hanmail.net (D.H.S)

**Keywords:** verproside, in vitro metabolism, UDP-glucuronosyltransferase, sulfotransferase

## Abstract

Verproside, an active iridoid glycoside component of Veronica species, such as *Pseudolysimachion rotundum* var. *subintegrum* and *Veronica anagallis-aquatica*, possesses anti-asthma, anti-inflammatory, anti-nociceptive, antioxidant, and cytostatic activities. Verproside is metabolized into nine metabolites in human hepatocytes: verproside glucuronides (**M1**, **M2**) via glucuronidation, verproside sulfate (**M3**) via sulfation, picroside II (**M4**) and isovanilloylcatalpol (**M5**) via *O*-methylation, **M4** glucuronide (**M6**) and **M4** sulfate (**M8**) via further glucuronidation and sulfation of **M4**, and **M5** glucuronide (**M7**) and **M5** sulfate (**M9**) via further glucuronidation and sulfation of **M5**. Drug-metabolizing enzymes responsible for verproside metabolism, including sulfotransferase (SULT) and UDP-glucuronosyltransferase (UGT), were characterized. The formation of verproside glucuronides (**M1**, **M2**), isovanilloylcatalpol glucuronide (**M7**), and picroside II glucuronide (**M6**) was catalyzed by commonly expressed UGT1A1 and UGT1A9 and gastrointestinal-specific UGT1A7, UGT1A8, and UGT1A10, consistent with the higher intrinsic clearance values for the formation of **M1**, **M2**, **M6**, and **M7** in human intestinal microsomes compared with those in liver microsomes. The formation of verproside sulfate (**M3**) and **M5** sulfate (**M9**) from verproside and isovanilloylcatalpol (**M5**), respectively, was catalyzed by SULT1A1. Metabolism of picroside II (**M4**) into **M4** sulfate (**M8**) was catalyzed by SULT1A1, SULT1E1, SULT1A2, SULT1A3, and SULT1C4. Based on these results, the pharmacokinetics of verproside may be affected by the co-administration of relevant UGT and SULT inhibitors or inducers.

## 1. Introduction

Verproside is a biologically active iridoid glycoside component of Pseudolysimachion rotundum var. subintegrum, Pseudolysimachionspurium, Pseudolysimachion longifolium, Veronica anagallis-aquatica, and Veronica ciliata [1,2,3,4]. Verproside exhibits anti-inflammatory [5,6,7], antioxidant [8], anti-nociceptive [6], anti-hepatocarcinoma [9], and anti-asthmatic activities. Verproside may be a good therapeutic candidate as an anti-asthma drug, blocking the tumor necrosis factor alpha (TNF-α)/nuclear factor kappa B (NF-κB) signaling pathway and reducing the levels of total immunoglobulin E, interleukin-4, and interleukin-13 [10,11]. There are a few reports on the pharmacokinetics and metabolism of verproside in rats [12,13], but the mechanisms underlying the in vitro and in vivo metabolism of verproside in animals and humans have not been reported. Verproside showed a short half-life (12.2–16.6 min), high systemic clearance (56.7–86.2 mL/min/kg), and low renal clearance (2.7–4.1 mL/min/kg) after intravenous injection (dose range 2–10 mg/kg) and very low bioavailability (<0.5%) after oral administration (dose range 20–100 mg/kg) in male Sprague Dawley rats, indicating the extensive metabolism of verproside [12]. In rats, verproside is metabolized into verproside glucuronides (**M1**, **M2**), verproside sulfates (**M3**, **M4**), picroside II (**M5**), **M5** glucuronide (**M7**), **M5** sulfate (**M9**), isovanilloylcatalpol (**M6**), **M6** glucuronide (**M8**), **M6** sulfate (**M10**), 3,4-dihydroxybenzoic acid (**M1**1), **M11** glucuronide (**M12**), **M11** sulfates (**M13**, **M14**), 3-methyoxy-4-hydroxybenzoic acid (**M15**), **M15** glucuronides (**M17**, **M18**), **M15** sulfate (**M20**), 3-hydroxy-4-methoxybenzoic acid (**M16**), **M16** glucuronide (**M19**), and **M16** sulfate (**M21**) via *O*-methylation, glucuronidation, sulfation, and hydrolysis. In total, 13 metabolites, **M1**–**M11**, **M13**, and **M14**, were identified in rat hepatocytes, and verproside sulfate (**M4**) was a major metabolite [13].

Metabolite identification and characterization of the enzymes responsible for the metabolism of drugs, such as cytochrome P450, UDP-glucuronosyltransferase (UGT), and sulfotransferase (SULT), can reveal potential drug–drug interactions and inter-individual variations in drug metabolism and pharmacokinetics [14,15,16]. It is important to establish the comparative metabolism and drug-metabolizing enzymes of verproside for a full characterization of its pharmacokinetics, pharmacodynamics, and toxicity. There has been no report on the in vitro or in vivo metabolic pathways of verproside in humans and characterization of the enzymes responsible for verproside metabolism.

Thus, the purpose of this study was to determine the metabolites of verproside formed from in vitro incubation of verproside with human hepatocytes, intestinal microsomes, and liver S9 fractions using liquid chromatography–high resolution quadrupole Orbitrap mass spectrometry (LC-HRMS). We also sought to characterize the UGT and SULT enzymes responsible for verproside metabolism using human cDNA-expressed UGT and SULT supersomes.

## 2. Results

### 2.1. In Vitro Metabolic Profile of Verproside in Human Hepatocytes

**Figure 1:**
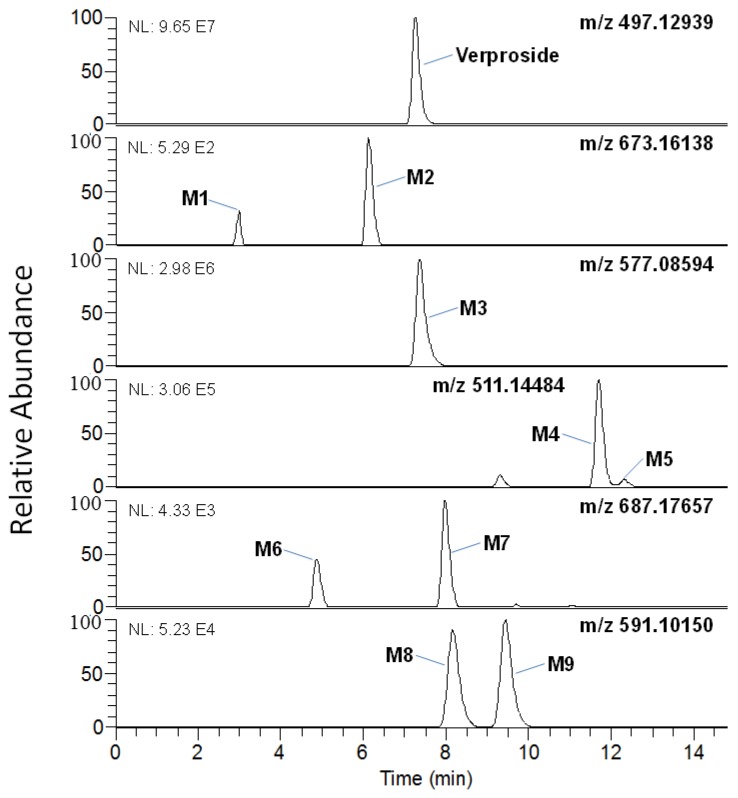
Chromatograms of the extracted ions of verproside and its metabolites in the liquid chromatography-mass spectrometry (LC-MS)analysis of the assays using human hepatocytes. The extracted ion chromatograms were reconstructed based on deprotonated molecular ions: *m*/*z* 497.12939 for verproside, 673.16138 for **M1** and **M2** (verproside glucuronides), 577.08594 for **M3** (verproside sulfate), 511.14484 for **M4** (picroside II) and **M5** (isovanilloylcatalpol), 687.17657 for **M6** (picroside II glucuronide) and **M7** (isovanilloylcatalpol glucuronide), and 591.10150 for **M8** (picroside II sulfate) and **M9** (isovanilloylcatalpol sulfate).

**Figure 2:**
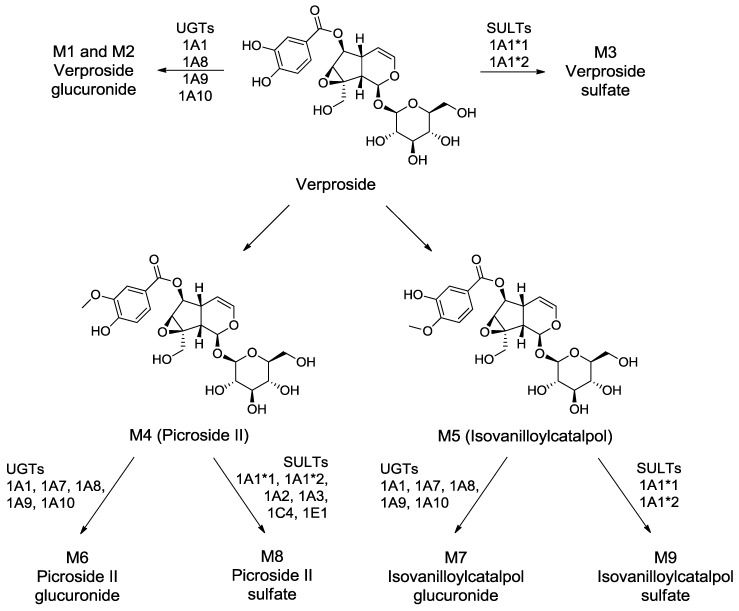
Possible in vitro metabolic pathways of verproside in human hepatocytes.

LC-HRMS analysis of the extracts after a 2-h incubation of verproside with human hepatocytes resulted in nine metabolites (**M1**–**M9**), along with unchanged verproside (Figure 1). The retention times and exact masses of the deprotonated molecular ion ([M − H]^−^) and diagnostic product ions of verproside and its nine metabolites (**M1**–**M9**) are shown in Table 1. **M1**–**M9** were commonly identified in human and rat hepatocytes [13]: verproside glucuronides (**M1**, **M2**), verproside sulfate (**M3**), picroside II (**M4**), isovanilloylcatalpol (**M5**), picroside II glucuronide (**M6**), isovanilloylcatalpol glucuronide (**M7**), picroside II sulfate (**M8**), and isovanilloylcatalpol sulfate (**M9**). **M4** and **M5** were identified as picroside II and isovanilloylcatalpol, respectively, by comparison with the accurate mass, retention time, and product scan spectra of the corresponding authentic standards. **M6** and **M8** were also identified after incubation of picroside II (**M4**) with human liver S9 fractions in the presence of uridine 5′-diphosphoglucuronic acid (UDPGA) and 3-phosphoadenosine- 5-phosphosulfate (PAPS). **M7** and **M9** were identified after incubation of isovanilloylcatalpol (**M5**) with human liver S9 fractions in the presence of UDPGA and PAPS. The exact sites for glucuronidation and sulfation of verproside, picroside II (**M4**), and isovanilloylcatalpol (**M5**) into **M1**–**M3** and **M6**–**M9** could not be identified because of the lack of authentic standards. Based on these results, the potential in vitro metabolic pathways of verproside in human hepatocytes are shown in Figure 2.

### 2.2. Characterization of Human UGT Enzymes Responsible for the Glucuronidation of Verproside, Isovanilloylcatalpol, and Picroside II

**Figure 3:**
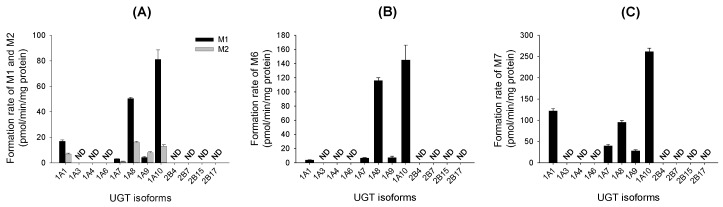
Formation of (**A**) verproside glucuronides (**M1**, **M2**) from 500 μM verproside; (**B**) picroside II glucuronide (**M6**) from 100 μM picroside II (**M4**); and (**C**) isovanilloylcatalpol glucuronide (**M7**) from 100 μM isovanilloylcatalpol (**M5**) in supersomes expressing recombinant human UDP-glucuronosyltransferase (UGT)1A1, UGT1A3, UGT1A4, UGT1A6, UGT1A7, UGT1A8, UGT1A9, UGT1A10, UGT2B4, UGT2B7, UGT2B15, and UGT2B17. ND: <0.67 pmol/min/mg protein for verposide. ND: <2.5 pmol/min/mg protein for picroside II and isovanilloylcatalpol. The data represent mean ± S.D. (*n* = 3).

Metabolite screening of verproside, picroside II, and isovanilloylcatalpol with 12 human cDNA-expressed UGTs showed that UGT1A1, UGT1A7, UGT1A8, UGT1A9, and UGT1A10 are involved in the formation of the verproside glucuronides **M1** and **M2** from verproside, the glucuronidation of picroside II (**M4**) to picroside II glucuronide (**M6**), and the glucuronidation of isovanilloylcatalpol (**M5**) to isovanilloylcatalpol glucuronide (**M7**; Figure 3). According to Li et al. [17], UGT1A7, UGT1A8, UGT1A9, and UGT1A10 catalyzed the glucuronidation of picroside II, with the highest contribution by UGT1A10.

**Figure 4:**
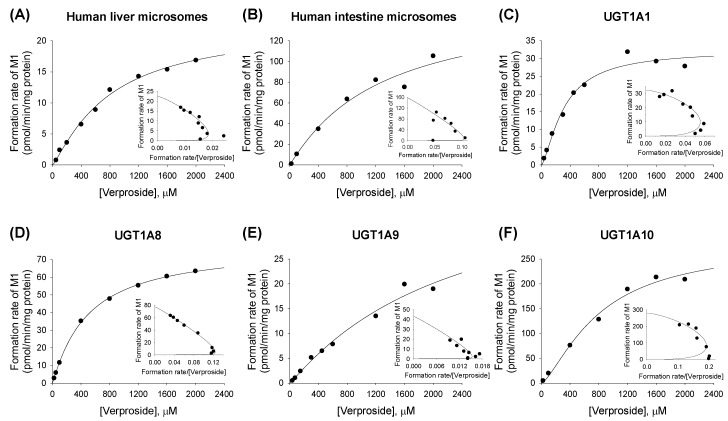
Michaelis–Menten plots for the formation of verproside glucuronide **M1** from verproside in (**A**) human liver microsomes; (**B**) human intestinal microsomes, and supersomes expressing recombinant human (**C**) UGT1A1; (**D**) UGT1A8; (**E**) UGT1A9; and (**F**) UGT1A10 enzymes. An Eadie–Hofstee plot is provided in the inset. The solid line is the curve fit line obtained using the Enzyme Kinetics program.

**Figure 5:**
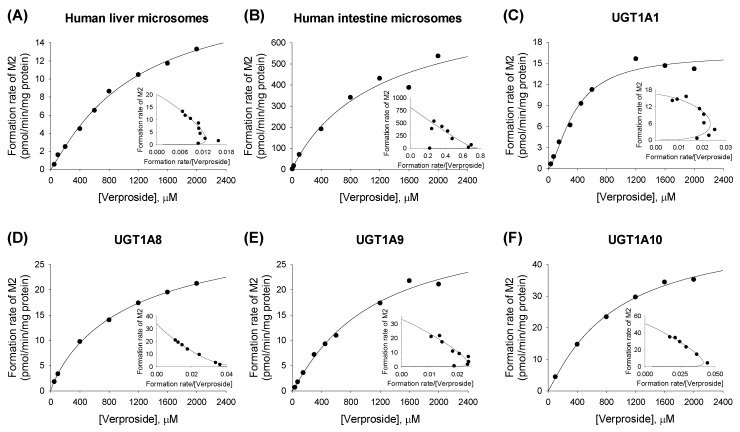
Michaelis-Menten plots for the formation of verproside glucuronide **M2** from verproside in (**A**) human liver microsomes; (**B**) human intestinal microsomes, and supersomes expressing recombinant human (**C**) UGT1A1; (**D**) UGT1A8; (**E**) UGT1A9; and (**F**) UGT1A10 enzymes. An Eadie-Hofstee plot is provided in the inset.

The formation rates of the two verproside glucuronides (**M1**, **M2**) from verproside demonstrated a better fit to the Hill equation for **M1** and **M2** compared with verproside concentrations in the presence of UDPGA in human liver microsomes, intestinal microsomes, and cDNA-expressed UGT1A1, UGT1A8, UGT1A9, and UGT1A10 enzymes (Figure 4 and Figure 5). Enzyme kinetic parameters, such as *K*_m_, *V*_max_, Hill coefficient (*n*), and intrinsic clearance (*Cl*_int_, *V*_max_/*K*_m_) values, for the formation of **M1** and **M2** from verproside in human liver microsomes, intestinal microsomes, and cDNA-expressed UGTs are summarized in Table 2.

**Figure 6:**
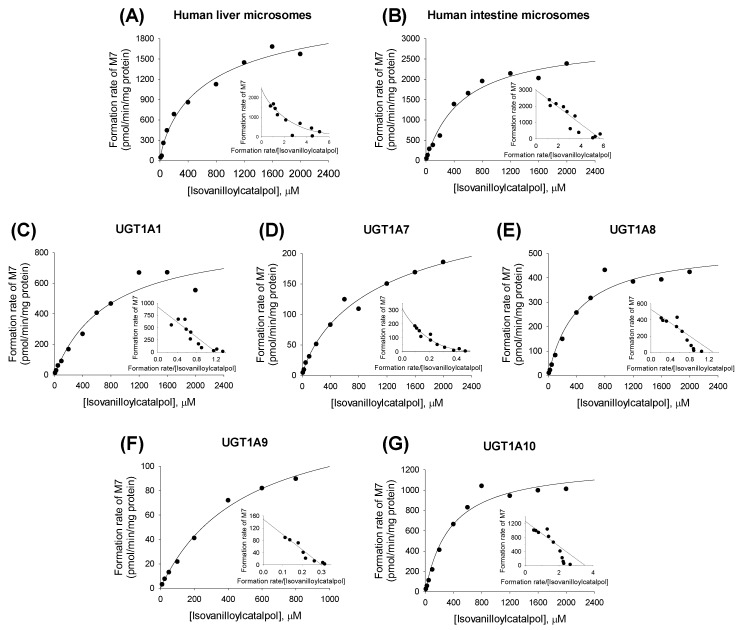
Michaelis–Menten plots for the formation of isovanilloylcatalpol glucuronide (**M7**) from isovanilloylcatalpol in (**A**) human liver microsomes, (**B**) human intestinal microsomes; and supersomes expressing recombinant human (**C**) UGT1A1; (**D**) UGT1A7; (**E**) UGT1A8; (**F**) UGT1A9; and (**G**) UGT1A10 enzymes. An Eadie–Hofstee plot is provided in the inset.

The formation of isovanilloylcatalpol glucuronide (**M7**) from isovanilloylcatalpol (**M5**) in human liver microsomes, intestinal microsomes, and cDNA-expressed UGT1A1, UGT1A7, UGT1A8, UGT1A9, and UGT1A10 enzymes followed single-enzyme kinetics and the Hill equation (Figure 6). Enzyme kinetic parameters, such as *K*_m_, *V*_max_, *n*, and *Cl*_int_ values, for the formation of **M7** from isovanilloylcatalpol in human liver microsomes, intestinal microsomes, and cDNA-expressed UGTs are summarized in Table 3.

### 2.3. Characterization of Human SULT Enzymes Responsible for Sulfation of Verproside, Isovanilloylcatalpol, and Picroside II

**Figure 7:**
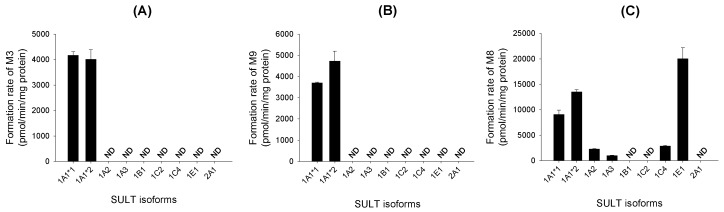
Formation of (**A**) verproside sulfate (**M3**) from 1.2 μM verproside; (**B**) isovanilloylcatalpol sulfate (**M9**) from 25 μM isovanilloylcatalpol (**M5**); and (**C**) picroside II sulfate (**M8**) from 25 μM picroside II (**M4**) in supersomes expressing recombinant human sulfotransferase (SULT)1A1*1, SULT1A1*2, SULT1A2, SULT1A3, SULT1B1, SULT1C2, SULT1C4, SULT1E1, and SULT2A1. Data represent the mean ± S.D. (*n* = 3). ND: <0.5 pmol.

Metabolite screening of verproside, picroside II (**M4**), and isovanilloylcatalpol (**M5**) with nine human cDNA-expressed SULTs showed that SULT1A1*1 and SULT1A1*2 were responsible for the sulfation of verproside and isovanilloylcatalpol (**M5**) into verproside sulfate (**M3**) and isovanilloylcatalpol sulfate (**M9**), respectively (Figure 7A,B). The sulfation of picroside II (**M4**) into picroside II sulfate (**M8**) was catalyzed by SULT1A1*1, SULT1A1*2, SULT1A2, SULT1A3, SULT1C4, and SULT1E1 (Figure 7C).

**Figure 8:**
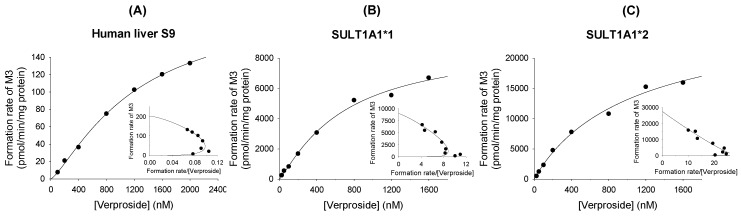
Michaelis-Menten plots of the formation of verproside sulfate (**M3**) from verproside in (**A**) human liver S9 fractions and (**B**) supersomes expressing recombinant human SULT1A1*1 and (**C**) SULT1A1*2. An Eadie–Hofstee plot is provided in the inset.

The formation of verproside sulfate (**M3**) from verproside followed Hill-equation kinetics in human liver S9 preparations and in cDNA-expressed SULT1A1*1 and SULT1A1*2 (Figure 8). The enzyme kinetic parameters for the formation of **M3** from verproside are shown in Table 4.

**Figure 9:**
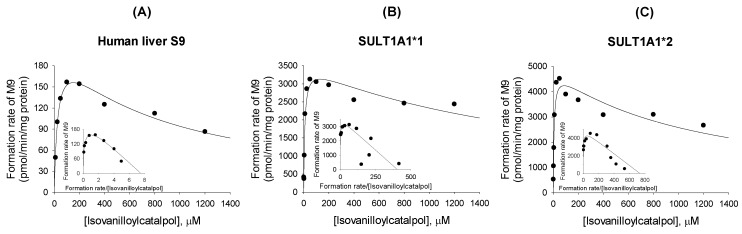
Michaelis–Menten plots of the formation of isovanilloylcatalpol sulfate (**M9**) from isovanilloylcatalpol (**M5**) in (**A**) human liver S9 fractions and (**B**) supersomes expressing recombinant human SULT1A1*1 and (**C**) SULT1A1*2. An Eadie–Hofstee plot is provided in the inset.

The formation of isovanilloylcatalpol sulfate (**M9**) from isovanilloylcatalpol (**M5**) followed enzyme-inhibition kinetics in human liver S9 and cDNA-expressed SULT1A1*1 and SULT1A1*2 (Figure 9). The enzyme kinetic parameters for the sulfation of isovanilloylcatalpol into **M9** are shown in Table 4.

**Figure 10:**
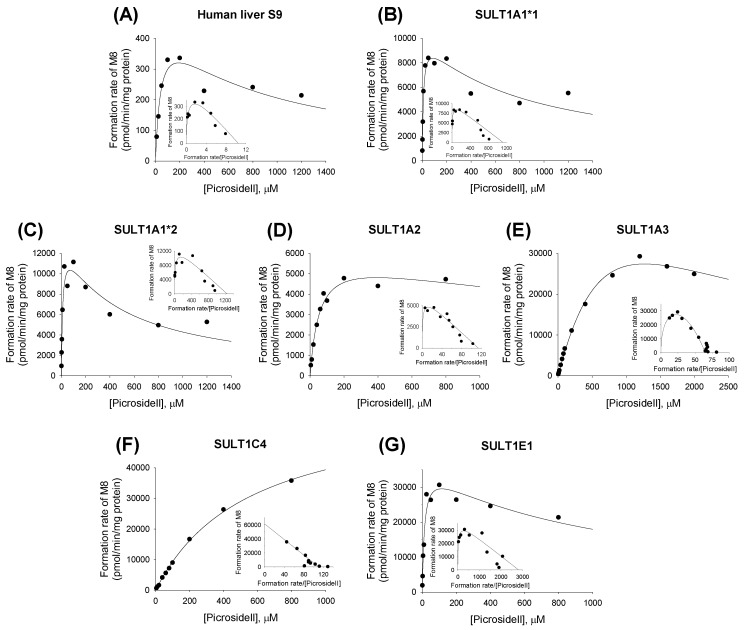
Michaelis–Menten plots of the formation of picroside II sulfate (**M8**) from picroside II (**M4**) in (**A**) human liver S9 fractions and (**B**) supersomes expressing recombinant human SULT1A1*1; (**C**) SULT1A1*2; (**D**) SULT1A2, (**E**) SULT1A3; (**F**) SULT1C4; and (**G**) SULT1E1. An Eadie–Hofstee plot is provided in the inset.

The formation of picroside II sulfate (**M8**) from picroside II (**M4**) followed single-enzyme or enzyme-inhibition kinetics in human liver S9 and cDNA-expressed SULTs (1A1*1, 1A1*2, 1A2, 1A3, 1C4, and 1E1) (Figure 10). The enzyme kinetic parameters for the sulfation of picroside II into **M8** are shown in Table 5.

## 3. Discussion

Verproside was metabolized into (i) verproside glucuronides (**M1**, **M2**) via glucuronidation, (ii) verproside sulfate (**M3**) via sulfation, (iii) picroside II (**M4**) and isovanilloylcatalpol (**M5**) via *O*-methylation, (iv) **M4** glucuronide (**M6**) and **M4** sulfate (**M8**) via further glucuronidation and sulfation of **M4**, and (v) **M5** glucuronide (**M7**) and **M5** sulfate (**M9**) via further glucuronidation and sulfation of **M5** in human hepatocytes, liver, and intestinal microsomes, and liver S9 fractions (Figure 1 and Figure 2). Picroside II (**M4**) and isovanilloylcatalpol (**M5**) were identified based on the retention time and product ions by comparison with the corresponding authentic standards. Because cathechol *O*-methyltransferase favors 3-*O-*methylation over 4-*O-*methylation, the amount of picroside II (**M4**) was more than that of isovanilloylcatalpol (**M5**) in human and rat hepatocytes [13]. **M6**–**M9** were also identified after incubation of picroside II (**M4**) and isovanilloylcatalpol (**M5**) with human liver microsomes and S9 preparations. In the rats, verproside was hydrolyzed to 3,4-dihydroxybenzoic acid, 3-methoxy-4-hydroxybenzoic acid, and 3-hydroxy-4-methoxybenzoic acid [13], but three hydrolysis metabolites were not detected in human hepatocytes.

Screening for 12 UGT enzymes showed that UGT1A1, UGT1A7, UGT1A8, UGT1A9, and UGT1A10 were responsible for the glucuronidation of verproside, picroside II, and isovanilloylcatalpol into verproside glucuronides (**M1**, **M2**), picroside II glucuronide (**M6**), and isovanilloylcatalpol glucuronide (**M7**; Figure 3). UGT1A1 and UGT1A9 are present in the liver and intestine, but UGTs 1A7, 1A8, and 1A10 are gastrointestinal-specific [18,19,20,21]. In human liver microsomes, UGT1A1 and UGT1A9 played major roles in the glucuronidation of verproside, picroside II, and isovanilloylcatalpol into **M1**, **M2**, **M6**, and **M7**. In human intestinal microsomes, UGT1A7, UGT1A8, and UGT1A10, as well as UGT1A1 and UGT1A9, catalyzed the glucuronidation of verproside, picroside II, and isovanilloylcatalpol. Thus, *Cl*_int_ values towards the formation of verproside glucuronides (**M1**, **M2**) and isovanilloylcatalpol glucuronide (**M7**) from verproside and isovanilloylcatalpol, respectively, in human intestinal microsomes were 1.9–37.0-fold higher than those in human liver microsomes: 0.126 versus 0.029 μL/min/mg protein for **M1**, 0.667 versus 0.018 μL/min/mg protein for **M2**, and 5.71 versus 3.02 μL/min/mg protein for **M7** (Table 2 and Table 3). According to Li et al. [17], the *Cl*_int_ value for picroside II glucuronidation was 12.3 μL/min/mg protein in human intestinal microsomes, but 2.3 μL/min/mg protein in human liver microsomes, with the highest contribution by UGT1A10. *Cl*_int_ values for verproside glucuronidation to **M1** and **M2** and isovanilloylcatalpol glucuronidation to **M7** by UGT1A10 were 1.3–18.4-fold higher than those by UGT1, UGT1A7, UGT1A8, and UGT1A9 (Table 2 and Table 3). UGT1A10 in human small intestine (17.3%) and colon (27.4%) samples showed a greater relative abundance in total UGT proteins than did UGT1A1 (12.6% and 2.8%), UGT1A9 (4.5% and 3.2%), UGT1A7 (1.6% and 1.8%), and UGT1A8 (0.8% and 1.5%) [21]. These results indicate that UGT1A10 may play the most prominent role, compared with UGT1A1, UGT1A9, UGT1A7, and UGT1A8, in the glucuronidation of verproside and isovanilloylcatalpol in human intestinal microsomes.

Screening of human cDNA-expressed SULTs showed that SULT1A1*1 and SULT1A1*2 were responsible for the sulfation of verproside and isovanilloylcatalpol (**M5**) into verproside sulfate (**M3**) and **M5** sulfate (**M9**), respectively, but the formation of picroside II sulfate (**M8**) from picroside II (**M4**) was catalyzed by human cDNA-expressed SULTs 1A1*1, 1A1*2, 1A2, 1A3, 1C4, and 1E1 (Figure 7). In human liver S9 fractions, verproside sulfation into **M3** (*K*_m_, 1.2 μM) showed 23.8- and 37.3-fold higher affinity than picroside II sulfation to **M8** (*K*_m_, 28.8 μM) and isovanilloylcatalpol sulfation into **M9** (*K*_m_, 45.1 μM). The hepatic metabolic activity of verproside sulfation into **M3** was higher than those of picroside II sulfation to **M8** (*Cl*_int_, 27.5 μL/min/mg protein) and isovanilloylcatalpol sulfation to **M9** (*Cl*_int_, 10.5 μL/min/mg protein).

Enzyme kinetics for verproside sulfation followed the Hill equation, but picroside II sulfation and isovanilloylcatalpol sulfation followed enzyme-inhibition and single-enzyme kinetics. The *K*_m_ values for verproside sulfation in SULT1A1*1 (0.69 μM) and SULT1A1*2 (1.10 μM) were similar to that in human liver S9 fractions (*K*_m_, 1.2 μM). The major hepatic SULT1A1*1 and SULT1A1*2 played predominant roles in verproside sulfation and isovanilloylcatalpol sulfation. In the formation of picroside II sulfate (**M8**) from picroside II, SULT1A1*1 (*K*_m_, 9.0 μM; *Cl*_int_, 1122 μL/min/mg protein), SULT1A1*2 (*K*_m_, 10.8 μM; *Cl*_int_, 1249 μL/min/mg protein), and SULT1E1 (*K*_m_, 12.9 μM; *Cl*_int_, 2810 μL/min/mg protein) showed higher affinity and metabolic activity than did SULT1A2 (*K*_m_, 54.0 μM; *Cl*_int_, 113.6 μL/min/mg protein), SULT1A3 (*K*_m_, 1271.7 μM; *Cl*_int_, 64.8 μL/min/mg protein), and SULT1C4 (*K*_m_, 553.1 μM; *Cl*_int_, 110.5 μL/min/mg protein). The total contents of five SULTs (1A1, 1A3, 1B1, 1E1, and 2A1) in tissue cytosol fractions ranked in the order of small intestine (7800 ng/mg cytosol protein) > liver (5960 ng/mg cytosol protein) > kidney (430 ng/mg cytosol protein) > lung (290 ng/mg cytosol protein) [22]. SULT1A1 was the major hepatic SULT (accounting for 53% of total hepatic SULT proteins) but was also present in substantial quantities in the small intestine (19% of total SULT protein) [22]. SULT1E1 was expressed at relatively low levels in the liver (6% of total SULT protein) and the small intestine (8% of total SULT protein) but was the most abundant enzyme in the lung (40% of total SULT protein). SULT1A3 was not detected in the liver but was a major enzyme in the small intestine (31% of total SULT protein). SULT1C4 expression has been reported to be highest in fetal lung and kidney, with lower expression in fetal heart and adult kidney, ovary, and spinal cord, using multi-tissue dot blot analyses [23]. Based on these results, SULT1A1 and SULT1E1 may be the major enzymes responsible for the metabolism of picroside II into picroside II sulfate, with minor contributions by SULT1A2, SULT1A3, and SULT1C4. These results suggest that there may be inter-individual variability in the pharmacokinetics of verproside as a result of the changes in the rates of metabolism based on wide inter-individual variability in the expression of SULT1A1 and SULT1E1 in humans [24,25]. SULT1A1 and SULT1E1 have been shown to be induced or inhibited by various drugs and chemicals [25,26,27]. Co-administration of drugs that can inhibit or induce SULT1A1 and SULT1E1 may alter verproside sulfation.

## 4. Materials and Methods 

### 4.1. Materials and Reagents

Verproside, picroside II, and isovanilloylcatalpol, isolated from *Pseudolysimachion rotundum* var. *subintegrum* as described previously [10], were gifts from KRIBB (Ochang, Korea). Alamethicin, PAPS, William′s E buffer, and UDPGA were obtained from Sigma-Aldrich (St. Louis, MO, USA). Pooled human liver microsomes, pooled human intestinal microsomes, pooled human liver S9 fractions, human cDNA-expressed UGT 1A1, 1A3, 1A4, 1A6, 1A7, 1A8, 1A9, 1A10, 2B4, 2B7, 2B15, and 2B17 supersomes, cryopreserved human hepatocytes, and cryopreserved hepatocyte purification kits were obtained from Corning Life Sciences (Woburn, MA, USA). Human cDNA-expressed SULT 1A1*1, 1A1*2, 1A2, 1A3, 1B1, 1C2, 1C4, 1E1, and 2A1 supersomes were obtained from Cypex Ltd. (Dundee, UK). 4-Methylumbelliferone was obtained from Toronto Research Chemicals (North York, ON, Canada). Acetonitrile and methanol (HPLC grade) were obtained from Burdick & Jackson, Inc. (Ulsan, South Korea). The other chemicals were of the highest quality available. ProteoMass LTQ/FT-hybrid ESI Positive mode Cal Mix (MSCAL5) and Negative mode Cal Mix (MSCAL6) for calibration of Q-Exactive MS were obtained from Supelco (Bellefonte, PA, USA).

### 4.2. In Vitro Metabolism of Verproside in Cryopreserved Human Hepatocytes

Cryopreserved human hepatocytes were purified and recovered using a high-viability cryohepatocyte recovery kit according to the manufacturer′s protocol. Purified human hepatocytes were resuspended in William′s E buffer to a final density of 0.8 × 10^6^ cells/mL [16]. A portion of the human hepatocyte suspension (62.5 μL; 5 × 10^4^ cells) and 62.5 μL of 400 μM verproside were added to a 96-well plate and incubated for 2 h at 37 °C in a CO_2_ incubator. Then, 500 μL methanol were added to the incubation mixture and centrifuged (13,000× g, 10 min, 4 °C). The supernatant (500 μL) was evaporated to dryness using a vacuum evaporator (Genovac, UK). The residue was dissolved in 100 μL of 5% methanol. An aliquot (5 μL) was injected into the LC-HRMS system to identify the metabolites of verproside.

### 4.3. Characterization of Human UGTs Responsible for the Glucuronidation of Verproside and its Metabolites, Picroside II, and Isovanilloylcatalpol

To screen for UGT enzymes responsible for the glucuronidation of verproside, picroside II, and isovanilloylcatalpol, 100 μL reaction mixtures containing human cDNA-expressed UGT 1A1, 1A3, 1A4, 1A6, 1A7, 1A8, 1A9, 1A10, 2B4, 2B7, 2B15, and 2B17 (10 μg protein), 2 mM UDPGA, 0.025 mg/mL alamethicin, and 500 μM verproside and its metabolites, 100 μM picroside II, or isovanilloylcatalpol, in 50 mM Tris buffer (pH 7.4) were incubated at 37 °C for 30 min in triplicate. The reaction was stopped by adding 100 μL 4-methylumbelliferone (internal standard, 30 ng/mL) in methanol. After vortex-mixing and centrifugation (13,000× *g*, 10 min, 4 °C), the supernatant (50 μL) was diluted with 50 μL water, and an aliquot (5 μL) was injected into the LC-HRMS system.

To evaluate the enzyme kinetic parameters for the formation of verproside glucuronides from verproside, various concentrations of verproside (final concentrations, 5–2000 μM) were incubated with 2 mM UDPGA, 0.025 mg/mL alamethicin, pooled human liver microsomes (50 μg protein), pooled human intestinal microsomes (30 μg protein), or human cDNA-expressed UGT1A1, UGT1A8, UGT1A9, or UGT1A10 supersomes (20 μg protein) at 37 °C for 30 min in duplicate. The reaction was stopped by adding 100 μL 4-methylumbelliferone (internal standard, 30 ng/mL) in methanol. After vortex-mixing and centrifugation (13,000× *g*, 10 min, 4 °C), the supernatant (50 μL) was diluted with 50 μL water, and an aliquot (5 μL) was injected into the LC-HRMS system.

To evaluate the enzyme kinetic parameters for the formation of isovanilloylcatalpol glucuronide from isovanilloylcatalpol, various concentrations of isovanilloylcatalpol (final concentrations, 10–2000 μM) were incubated with 2 mM UDPGA, 0.025 mg/mL alamethicin, pooled human liver microsomes (50 μg protein), pooled human intestinal microsomes (15 μg protein), or human cDNA-expressed UGT1A1, UGT1A7, UGT1A8, UGT1A9, or UGT1A10 supersomes (10 μg protein) at 37 °C for 20 min in duplicate. The reaction was stopped by adding 100 μL 4-methylumbelliferone (internal standard, 30 ng/mL) in methanol. After vortex-mixing and centrifugation (13,000× *g*, 10 min, 4 °C), the supernatant (50 μL) was diluted with 50 μL water, and an aliquot (5 μL) was injected into the LC-HRMS system.

Authentic standards of verproside glucuronides (**M1**, **M2**), picroside II glucuronide (**M6**), and isovanilloylcatalpol glucuronide (**M7**) were not available. Thus, **M1**, **M2**, **M6**, and **M7** were determined using the verproside, picroside II, and isovanilloylcatalpol calibration curves, respectively. Consequently, a limitation exists in the accurate interpretation of the enzyme kinetic data for **M1**, **M2**, **M6**, and **M7** because each sensitivity of the metabolites was hypothesized to be the same as the corresponding substrate.

### 4.4. Characterization of Human SULTs Responsible for the Sulfation of Verproside and its Metabolites, Picroside II, and Isovanilloylcatalpol

To screen SULT enzymes responsible for the sulfation of verproside, picroside II, and isovanilloylcatalpol, 100 μL reaction mixture containing 20 μM PAPS, 10 mM dithiothreitol, 5 mM magnesium chloride, 50 mM phosphate buffer (pH 7.4), verproside (1.2 μM), picroside II (25 μM), or isovanilloylcatalpol (25 μM), and human liver S9 fractions (10 μg protein) or human cDNA-expressed SULTs 1A1*1, 1A1*2, 1A2, 1A3, 2A1, 1B1, 1C2, 1C4, or 1E1 supersomes (1A1*1: 0.25; 1A1*2: 0.5; 1A2: 0.25; 1A3: 0.2; 2A1: 1.25; 1B1: 0.5; 1C2: 2.5; 1C4: 0.1; 1E1: 0.2 μg protein) were incubated at 37 °C for 5 min in triplicate. The reaction was stopped by adding 50 μL 4-methylumbelliferone in methanol (30 ng/mL). After vortex-mixing and centrifugation, 50 μL of the supernatant was diluted with 50 μL deionized water. The mixture was transferred to an injection vial, and then an aliquot (5 μL) was injected into the LC-HRMS system.

In the enzyme kinetic experiments for the metabolism of verproside into verproside sulfate, various concentrations of verproside (10–2000 nM) were incubated with pooled human liver S9 fractions (10 μg protein), human cDNA-expressed SULT1A1*1 (0.25 μg protein) or SULT1A1*2 (0.5 μg protein), 20 μM PAPS, 10 mM dithiothreitol, and 5 mM magnesium chloride at 37 °C for 5 min in duplicate. In the kinetic experiments for the sulfation of isovanilloylcatalpol into isovanilloylcatalpol sulfate, various concentrations of isovanilloylcatalpol (1–1200 μM) were incubated with pooled human liver S9 fractions (20 μg protein), human cDNA-expressed SULT1A1*1 or SULT1A1*2 (0.4 μg protein), 20 μM PAPS, 10 mM dithiothreitol, and 5 mM magnesium chloride at 37 °C for 6 min in duplicate. To evaluate the enzyme kinetic parameters for the sulfation of picroside II into picroside II sulfate, various concentrations of picroside II (1–2000 μM) were incubated with pooled human liver S9 fractions (20 μg protein) and human cDNA-expressed SULT 1A1*1 (0.4 μg protein), 1A2 (0.4 μg protein), 1A3 (0.4 μg protein), 1C4 (0.4 μg protein), or 1E1 (0.2 μg protein), 20 μM PAPS, 10 mM dithiothreitol, and 5 mM magnesium chloride at 37 °C for 6 min in duplicate. The reaction was stopped by adding 100 μL 4-methylumbelliferone (internal standard, 30 ng/mL) in methanol. After vortex-mixing and centrifugation (13,000× *g*, 10 min, 4 °C), the supernatant (50 μL) was diluted with 50 μL water, and an aliquot (5 μL) was injected into the LC-HRMS system.

Authentic standards of verproside sulfate (**M3**), picroside II sulfate (**M8**), and isovanilloylcatalpol sulfate (**M9**) were not available. Thus, **M3**, **M8**, and **M9** were determined using the verproside, picroside II, and isovanilloylcatalpol calibration curves, respectively. Consequently, a limitation exists in the accurate interpretation of the enzyme kinetic data for **M3**, **M8**, and **M9** because each sensitivity of the metabolites was hypothesized to be the same as the corresponding substrate.

### 4.5. LC-HRMS Analysis of Verproside and its Metabolites

Verproside and its metabolites were separated using the Q-Exactive Orbitrap mass spectrometer coupled with the Accela UPLC system (Thermo Scientific, San Jose, CA, USA) using our LC-HRMS method described previously [13]. The separation was performed on a Halo C18 column (2.7 μm, 2.1 mm i.d. × 100 mm; Advanced Materials Technology, Wilmington, DE, USA) using a gradient elution of 5% methanol in ammonium formate (1 mM, pH 3.1; mobile phase A) and methanol (mobile phase B) at a flow rate of 0.5 mL/min: 5% mobile phase B for 3 min, 5% to 25% mobile phase B in 6 min, 25% to 90% mobile phase B in 0.4 min, 90% mobile phase B for 4.5 min, 90% to 5% mobile phase B in 0.4 min, and 5% mobile phase B for 4 min. The column and autosampler were maintained at 40 °C and 6 °C, respectively. Accurate mass measurements for verproside and its metabolites were performed by electrospray ionization in negative mode using the following electrospray source settings: ion transfer capillary temperature, 330 °C; needle spray voltage, −3000 V; capillary voltage, −40 V; nitrogen sheath gas, 50 arbitrary units; auxiliary gas, 15 arbitrary units. Automatic gain control and resolution settings were scaled to 1,000,000 and 70,000, respectively. MS data were acquired using an external calibration in the scan range of *m*/*z* 100–900 and were processed using the Xcalibur software (ver. 2.2; Thermo Scientific). Higher-energy collision dissociation with nitrogen gas and a collision energy of 25 eV were used to obtain product ion spectra for verproside and its metabolites. The proposed structures for the product ions of verproside and its metabolites were determined using the Mass Frontier software (ver. 6.0; HighChem Ltd., Bratislava, Slovakia).

Chromatograms were extracted using 5 ppm mass accuracy and extracted ion-monitoring mode was used for quantification: *m*/*z* 497.12939 for verproside, *m*/*z* 673.16138 for verproside glucuronides, *m*/*z* 577.08594 for verproside sulfate, *m*/*z* 511.14484 for picroside II and isovanilloylcatalpol, *m*/*z* 591.10150 for picroside II sulfate and isovanilloylcatalpol sulfate, *m*/*z* 687.17657 for picroside II glucuronide and isovanilloylcatalpol glucuronide, and *m*/*z* 175.03970 for 4-methylumbelliferone (internal standard). Peak areas for all components were integrated automatically using the Xcalibur software. The calibration curves for verproside, picroside II, and isovanilloylcatalpol were linear over the concentration range from 0.5–200 pmol.

### 4.6. Data Analysis

Results are presented as the mean of two determinations obtained each from human intestinal microsomes, liver microsomes, liver S9 fractions, UGTs, and SULTs. The apparent kinetic parameters (*K*_m_ and *V*_max_) were determined by fitting the single enzyme model [*V* = *V*_max_ × S/(*K*_m_ + S)], the Hill equation [*V* = *V*_max_ × S^n^/(*K*_m_^n^ + *S*^n^)], or the substrate-inhibition model [*V* = *V*_max_/(1 + *K*_m_/S) + S/*K*_i_)] using the Enzyme Kinetics program (ver. 1.3; Systat Software Inc., San Jose, CA, USA). In the equations above, *V* is the velocity of the reaction at the substrate concentration [S], *V*_max_ is the maximum velocity, *K*_m_ is the substrate concentration at which the reaction velocity is 50% of *V*_max_, and *n* is the Hill coefficient. The intrinsic clearance (*Cl*_int_) of the in vitro incubation was calculated as *V*_max_/*K*_m_.

## 5. Conclusions

Verproside was metabolized into nine metabolites in human hepatocytes: verproside glucuronides (**M1**, **M2**) via glucuronidation, verproside sulfate (**M3**) via sulfation, picroside II (**M4**) and isovanilloylcatalpol (**M5**) via *O*-methylation, **M4** glucuronide (**M6**) and **M4** sulfate (**M8**) via further glucuronidation and sulfation of **M4**, and **M5** glucuronide (**M7**) and **M5** sulfate (**M9**) via further glucuronidation and sulfation of **M5**. The formation of verproside sulfate (**M3**) and **M5** sulfate (**M9**) from verproside and isovanilloylcatalpol (**M5**), respectively, was catalyzed by SULT1A1. Metabolism of picroside II (**M4**) into **M4** sulfate (**M8**) was catalyzed by SULT1A1, SULT1E1, SULT1A2, SULT1A3, and SULT1C4. UGT1A1, UGT1A9, and gastrointestinal-specific UGT1A7, UGT1A8, and UGT1A10 played predominant roles in the formation of verproside glucuronides (**M1**, **M2**), isovanilloylcatalpol glucuronide (**M7**), and picroside II glucuronide (**M6**), with the highest metabolic activity observed for gastrointestinal-specific UGT1A10. These results suggest that the pharmacokinetics of verproside may be affected by co-administration of the relevant UGT and SULT inhibitors or inducers.

## Figures and Tables

**Table 1:** Accurate masses of the deprotonated molecular ion ([M − H]^−^) and product ions and the retention times of verproside and its metabolites identified in human hepatocytes.

Code	Compound Name	Retention Time (min)	Detected ([M − H]^−^) (*m*/*z*)	Error (ppm)	Product Ions (*m*/*z*)
	Verproside	7.25	497.12939	−1.5	355.0772, 221.0450, 153.0184
**M1**	Verproside glucuronide	2.99	673.16138	−0.9	497.1301, 335.0773, 221.0450, 175.0241
**M2**	Verproside glucuronide	6.11	673.16138	−0.9	497.1301, 335.0773, 221.0450, 175.0241
**M3**	Verproside sulfate	7.36	577.08594	−1.6	497.1301, 335.0773, 221.0451, 153.0184
**M4**	Picroside II	11.70	511.14484	−1.7	349.0928, 235.0608, 167.0341
**M5**	Isovanilloylcatalpol	12.32	511.14484	−1.7	349.0928, 235.0608, 167.0341
**M6**	Picroside II glucuronide	4.87	687.17657	−1.7	511.1458, 349.0930, 235.0609, 167.0342
**M7**	Isovanilloylcatalpol glucuronide	7.98	687.17657	−1.7	511.1458, 349.0930, 235.0609, 167.0342
**M8**	Picroside II sulfate	8.16	591.10150	−1.5	511.1457, 349.0927, 235.0609, 167.0341
**M9**	Isovanilloylcatalpol sulfate	9.44	591.10150	−1.5	511.1457, 349.0927, 235.0609, 167.0341

**Table 2:** Enzyme kinetic parameters for the formation of verproside glucuronides, **M1** and **M2**, from verproside in human liver microsomes, intestinal microsomes, and cDNA-expressed UDP-glucuronosyltransferase (UGT)1A1, UGT1A8, UGT1A9, and UGT1A10 supersomes.

Enzymes	M1 Formation				M2 Formation			
	*K*_m_ (μM)	*V*_max_	*n*	*Cl*_int_	*K*_m_ (μM)	*V*_max_	*n*	*Cl*_int_
Human Liver Microsomes	791.3 (174.9)	22.6 (2.5)	1.2	0.029	1109.9 (265.9)	20.0 (2.5)	1.1	0.018
Human Intestinal Microsomes	1251.8 (1608.4)	159.4 (100.3)	1.1	0.126	1215.2 (1460.9)	810.2 (449.0)	1.0	0.667
UGT1A1	316.5 (54.2)	32.4 (2.6)	1.4	0.102	363.7 (53.1)	16.3 (1.2)	1.5	0.045
UGT1A8	492.3 (30.1)	77.4 (1.9)	1.1	0.157	1158.7 (159.4)	34.2 (2.0)	0.9	0.030
UGT1A9	2272.1 (2873.8)	43.2 (31.4)	1.1	0.019	1052.5 (440.9)	33.0 (6.8)	1.1	0.031
UGT1A10	802.3 (266.4)	280.5 (54.6)	1.4	0.350	894.1 (229.5)	50.9 (6.4)	1.1	0.057

*V*_max_: pmol/min/mg protein, *Cl*_int_ (*V*_max_/*K*_m_): μL/min/mg protein, *n*: Hill coefficient. Standard errors are presented in parentheses. The amounts of the verproside glucuronides, **M1** and **M2**, were determined using the calibration curve for verproside.

**Table 3:** Enzyme kinetic parameters for the formation of isovanilloylcatalpol glucuronide (**M7**) from isovanilloylcatalpol (**M5**) in human liver microsomes, intestinal microsomes, and cDNA-expressed UGT enzymes.

Enzymes	*K*_m_ (μM)	*V*_max_	*n*	*Cl*_int_
Human liver microsomes	818.1 (591.4)	2470.7 (633.1)	0.7847	3.02
Human intestinal microsomes	520.9 (96.6)	2974.3 (201.4)	-	5.71
UGT1A1	768.8 (250.7)	919.6 (128.0)	-	1.20
UGT1A7	1312.6 (939.6)	312.9 (92.3)	0.8375	0.238
UGT1A8	412.4 (98.6)	529.3 (42.2)	-	1.28
UGT1A9	495.7 (77.9)	149.2 (11.7)	-	0.301
UGT1A10	358.5 (74.0)	1265.6 (82.5)	-	3.53

*V*_max_: pmol/min/mg protein, *Cl*_int_ (*V*_max_/*K*_m_): μL/min/mg protein, *n*: Hill coefficient. Standard errors are presented in parentheses. The amounts of isovanilloylcatalpol glucuronide was determined using the calibration curve for isovanilloylcatalpol.

**Table 4:** Enzyme kinetic parameters for the formation of verproside sulfate (**M3**) from verproside and isovanilloylcatalpol sulfate (**M9**) from isovanilloylcatalpol (**M5**) in human liver S9 fractions and cDNA-expressed SULT1A1*1 and SULT1A1*2 supersomes.

Enzymes	M3 Formation				M9 Formation			
	*K*_m_ (μM)	*V*_max_	*n*	*Cl*_int_	*K*_m_ (μM)	*V*_max_	*K*_i_ (μM)	*Cl*_int_
Human liver S9	1.21 (0.19)	203.3 (19.3)	1.3	168.0	28.8(5.3)	215.6 (15.4)	792.4 (147.5)	27.5
SULT1A1*1	0.69 (0.23)	9019.9 (1457.6)	1.2	13,131	8.8 (2.3)	3550.7 (268.7)	1938.6(726.7)	380.8
SULT1A1*2	1.10 (0.66)	27,610.0 (7798.5)	1.0	25,039	6.7 (1.9)	4889.2 (395.7)	1127.5 (365.0)	729.7

*V*_max_: pmol/min/mg protein; *Cl*_int_: μL/min/mg protein, *n*: Hill coefficient. Standard errors are presented in parentheses. The amounts of verproside sulfate and isovanilloylcatalpol sulfate were determined using the calibration curves for verproside and isovanilloylcatalpol, respectively.

**Table 5:** Enzyme kinetic parameters for the formation of picroside II sulfate (**M8**) from picroside II (**M4**) in human liver S9 fractions and human cDNA-expressed SULT enzymes.

Enzymes	*K*_m_ (μM)	*V*_max_	*K*_i_ (μM)	*Cl*_int_
Human liver S9	45.1 (22.0)	472.2 (101.9)	799.8 (417.1)	10.5
SULT1A1*1	9.0 (2.6)	10,100 (921)	844.4 (263.0)	1122
SULT1A1*2	10.8 (3.8)	13,490 (1710)	468.9 (163.9)	1249
SULT1A2	54.0 (13.4)	6134.5 (688.0)	2859.3 (1854.2)	113.6
SULT1A3	1271.7 (423.6)	82,370 (21746)	1265.9 (551.3)	64.8
SULT1C4	553.1 (33.4)	61,140 (2036)	-	110.5
SULT1E1	12.9 (3.4)	36,250 (3274)	995.3 (356.9)	2810

*Vmax*: pmol/min/mg protein; *Cl*_int_: μL/min/mg protein. Standard errors are presented in parentheses. The amounts of picroside II sulfate was determined using the calibration curve for picroside II.
